# The time-course of feature-based attention effects dissociated from temporal expectation and target-related processes

**DOI:** 10.1038/s41598-022-10687-x

**Published:** 2022-04-28

**Authors:** Denise Moerel, Tijl Grootswagers, Amanda K. Robinson, Sophia M. Shatek, Alexandra Woolgar, Thomas A. Carlson, Anina N. Rich

**Affiliations:** 1 School of Psychological Sciences, Macquarie University, Sydney, Australia; 2 Perception in Action Research Centre, Macquarie University, Sydney, Australia; 3 The MARCS Institute for Brain, Behaviour and Development, Western Sydney University, Sydney, Australia; 4 School of Psychology, University of Sydney, Sydney, Australia; 5 Queensland Brain Institute, The University of Queensland, Brisbane, Australia; 6 MRC Cognition and Brain Sciences Unit, University of Cambridge, Cambridge, UK; 7 Centre for Elite Performance, Expertise and Training, Macquarie University, Sydney, Australia

**Keywords:** Cognitive neuroscience, Attention, Perception

## Abstract

Selective attention prioritises relevant information amongst competing sensory input. Time-resolved electrophysiological studies have shown stronger representation of attended compared to unattended stimuli, which has been interpreted as an effect of attention on information coding. However, because attention is often manipulated by making only the attended stimulus a target to be remembered and/or responded to, many reported attention effects have been confounded with target-related processes such as visual short-term memory or decision-making. In addition, attention effects could be influenced by temporal expectation about when something is likely to happen. The aim of this study was to investigate the dynamic effect of attention on visual processing using multivariate pattern analysis of electroencephalography (EEG) data, while (1) controlling for target-related confounds, and (2) directly investigating the influence of temporal expectation. Participants viewed rapid sequences of overlaid oriented grating pairs while detecting a “target” grating of a particular orientation. We manipulated attention, one grating was attended and the other ignored (cued by colour), and temporal expectation, with stimulus onset timing either predictable or not. We controlled for target-related processing confounds by only analysing non-target trials. Both attended and ignored gratings were initially coded equally in the pattern of responses across EEG sensors. An effect of attention, with preferential coding of the attended stimulus, emerged approximately 230 ms after stimulus onset. This attention effect occurred even when controlling for target-related processing confounds, and regardless of stimulus onset expectation. These results provide insight into the effect of feature-based attention on the dynamic processing of competing visual information.

## Introduction

To interact effectively in our environment, we need to select relevant information from the continuous stream of incoming visual information. It is important to understand how selective attention influences different stages of perceptual processing, to gain insight into the mechanisms by which the brain prioritises the important information. Selective attention has been shown to involve a network of regions, involving frontal and parietal cortex^1–9^, which has a top-down influence on processing in sensory cortex and thalamus^10–16^. Single neurons in this network have been suggested to show adaptive coding^17,18^ or mixed selectivity^19,20^, as they adjust their tuning profiles to code for the features that are relevant for the current task demands. This network may in turn bias the response in regions such as visual cortex^21^, boosting representations of attended features and suppressing those of unattended features. At the neuronal level, attention has been shown to affect the noise that is shared across the neuronal population, by reducing interneuronal correlations^22^. In addition, attention has been shown to reduce the trial-to-trial variability of the neuronal response^23^. Time-resolved electrophysiological methods such as electroencephalography (EEG) and magnetoencephalography (MEG) combined with multivariate decoding methods are well suited to capture these changes in patterns of neural population responses with high temporal resolution. This method has been used to study which stage of perceptual processing is affected by selective attention. Manipulations of spatial attention result in a stronger neural response to stimuli at a cued compared to an uncued location, starting around 80–100 ms after stimulus onset^24–29^. The time-course of feature-selective attention is thought to be slower compared to spatial attention, with stronger neural responses to the cued compared to the uncued feature around 100–150 ms after stimulus onset^30–33^, but see^34^). In addition, several studies have used multivariate decoding of time-resolved neural data to show that the coding of cued information is sustained over time, whereas uncued information is represented only transiently and is not sustained over time^35–39^. Although these previous studies have interpreted preferential coding of cued over uncued information as an effect of attention, their findings could be influenced by two other potential processes: target-related processes and expectation effects.

Attention is often manipulated by making the cued stimulus a target, while the uncued stimulus is not. This means that target-related processes might influence neural responses—for the cued/target stimulus only—and this could drive differences in coding of cued and uncued information that are being attributed to attention. Here, we define target-related processes as all processes other than attention that might occur when responding to a target stimulus. Specifically, we consider visual short-term memory and decision-making as two target related processes that could confound attention findings. First, the cued target stimulus is often kept in visual short-term memory until the participant can make a decision. Second, the participant usually makes decisions about the cued stimulus, but not about the uncued stimulus. Critically, the stimulus feature (e.g., object shape) that is kept in visual short-term memory and used to make a classification decision is usually the same stimulus feature that is used in the decoding analysis. Either of these target-related processes could contribute to stronger information coding for the cued compared to uncued stimuli. A few studies have separated effects of attention from target-related processes by comparing the neural response to cued non-targets with uncued non-targets using either forward encoding models^40^ or pattern classification^37,39^. One such study^40^ showed enhanced coding of stimuli that were task-relevant in one session but not in the other session. The authors did not analyse the neural response to targets, but rather to all non-target stimuli in the sequence, avoiding target-related confounds. However, the attended and unattended items were not present at the same time, but across different sessions, which means these results might not generalise to attention effects when a distractor must be suppressed. Another study^37^ presented stimuli at in the same task context, while also only analysing the neural response to non-targets. The results showed enhanced of coding of the visual information that was currently relevant for the task relative to irrelevant visual information. However, the authors used a task which required holding only the cued information in visual short-term memory, thus again requiring processes additional to attention. In our previous work, we showed enhanced coding of the cued compared to the uncued orientations^39^, when followed by an orthogonal decision that was separated in time from the stimulus. However, here too participants had to keep the cued orientation in mind until the decision screen, which means that these results could also be partly driven by visual short-term memory. It is still unclear whether effects of attention on visual processing occur when we control for the influence of possible target-related processes.

In addition to target-related processes, the effects of selective attention on stimulus coding could be influenced by expectations about when a stimulus is likely to occur in time. Attention and temporal expectation could work together in an additive or interactive way to influence the processing of visual information^40–42^. Here, we use the term *attention* to refer to the mechanism that prioritises stimulus processing based on motivational relevance, in line with Summerfield and Egner^43^. In contrast, *expectation* refers to the mechanism that constrains visual interpretation based on prior likelihood^43^. Specifically, *temporal expectation* refers to expectations about *when* something is likely to happen. Single unit studies in non-human primates have shown that temporal expectation can modulate the neuronal firing rate, with higher firing rates for relevant inputs at expected moments, for neurons in inferotemporal cortex^44^ and V1^45^. This modulation could ultimately lead to improve the quality of the sensory information^46^. Temporal expectation has been experimentally manipulated through different temporal structures, such as through the use of explicit cues, or implicitly learned temporal rhythms^47^. Here we focus on implicitly learned rhythms of visual stimulus onset, as the explicit cues used to manipulate expectation can be very similar to those used in attention paradigms (see^48^ for a discussion). Some studies have found evidence of an effect of temporal expectation, as manipulated through rhythms, on the efficiency of early visual processing^49,50^. These studies found increased contrast sensitivity for visual stimuli presented at fixed compared to irregular intervals^49,50^, which was associated with increased phase entrainment of 1-4 Hz oscillations^49^. In addition, this entrainment was related to the behavioural discriminability of targets presented at a regular interval^49^. Several studies have directly investigated the interaction between attention and expectation, but most of these studies focus on either spatial or stimulus feature expectations, that is, expectations about *where* or *what*, rather than *when* something will occur. These studies have found interactions between attention and expectation^40–42^, as well as additive effects^42^. There is currently no consensus on whether temporal expectation is a possible confound in attention research, as little is known about the interaction between attention and implicitly learned temporal expectations. The lack of consensus is apparent from the variability in whether or not attention researchers make sure the stimulus onset is unpredictable to avoid temporal expectation confounds.

In this study, we investigated the time-course of the effect of attention on the coding of visual information in the brain. We compared the coding of attended and unattended visual stimuli that were presented simultaneously in time and space, while (1) making sure the pattern classification could not be driven by target-related processing, and (2) directly investigating the influence of temporal expectation. We recorded EEG data while participants performed a target detection task on sequences of central stimuli comprised of overlaid oriented blue and orange lines. We manipulated feature-based attention using colour: the participant was cued to attend to one colour orientation stimulus with another colour denoting a distractor, for each sequence. We used multivariate pattern analysis (MVPA) to compare the orientation coding of the same stimuli when they were attended or not. We controlled for effects of target-related processes by using a target detection task, where participants did not have to retain the cued stimulus orientation over a delay period, and analysing only the responses to non-target stimuli. To examine the influence of temporal expectation on attention effects, we compared the orientation coding of stimuli presented at a constant (predictable) stimulus onset with that from varied (unpredictable) stimulus onset. Our results showed preferential coding of the cued compared to the uncued information emerging from about 230 ms after stimulus onset. This difference is likely driven by the prioritisation of the task-relevant information through attention, as our manipulations made sure that target-related processes could not drive the classification, and it did not interact with temporal expectation.

## Methods

### Participants

Twenty healthy adults participated in this study (12 female/8 male; 19 right-handed/1 left-handed; mean age = 23.80 years; age range = 18–59 years). All participants reported normal or corrected-to-normal vision and normal colour vision. Participants were recruited from the University of Sydney and received $40 AUD for their participation. The study was approved by the University of Sydney ethics committee and informed consent was obtained from all participants. All methods were performed in accordance with the relevant guidelines and regulations of the University of Sydney ethics committee and the Declaration of Helsinki.

### Stimuli and design

**Figure 1:**
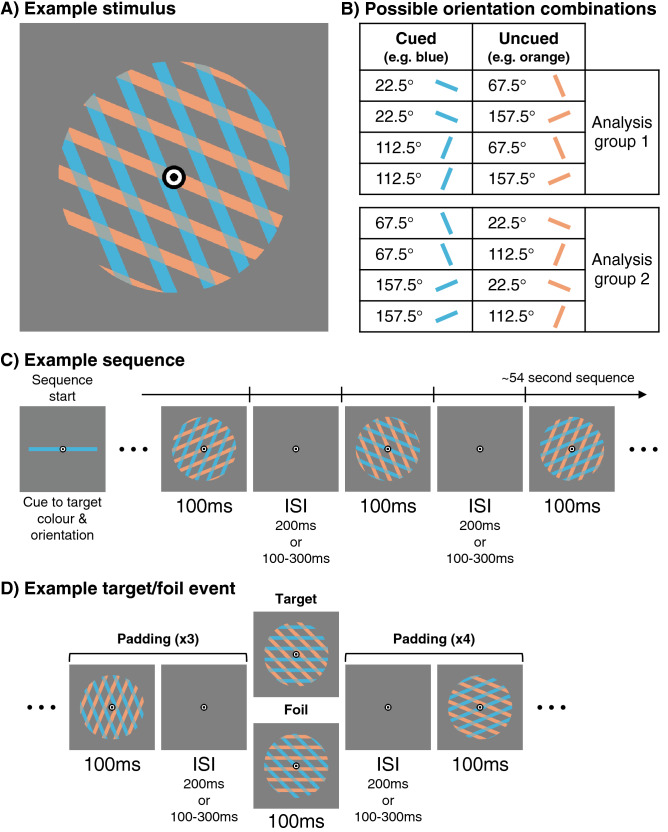
Experimental design. (**A**) The stimuli consisted of blue and orange oriented lines, overlaid at fixation. (**B**) There were 4 possible line orientations: 22.5°, 67.5° 112.5°, 157.5°. The blue and orange lines were always rotated 45° from each other, resulting in 8 possible combinations of orientations. We made sure the cued and uncued orientations were orthogonal in the decoding analysis by dividing the orientations into 2 analysis groups, labelled Analysis group 1 and 2. The decoding was done within group. For example, for the cued orientation in Analysis group 1 we decoded 22.5° vs. 112.5°. The decoding of the uncued orientation was completed in the same way. For example, for the uncued orientation in Analysis group 1 we decoded 67.5° vs. 157.5°. Note that for each cued orientation, both uncued orientations occur equally often and vice versa, which means that the other feature is not informative to the decoding analysis and cannot be used by the classifier. (**C**) Shows an example of part of a sequence. At the start of each sequence, participants saw the target colour and orientation for that sequence until they pressed a key to start. Stimuli were presented for 100 ms. Each stimulus consisted of lines of the cued and uncued colour. The ISI for the sequence was either constant at 200 ms or varied between 100 and 300 ms. (**D**) Shows an example of a target or foil event. In this example, the target is horizontal blue lines. The target is shown at the top (cued blue target orientation), and the foil at the bottom (uncued orange target orientation). Participants had to respond to targets but not to foils. The target and foil trials, along with 3 padding trials before and 4 padding trials after each target or foil, were not used in the analysis. Only the non-target stimuli were used in the analysis.

The stimuli consisted of blue (RGB = 239, 159, 115) and orange (RGB = 72, 179, 217) oriented lines, overlaid at fixation, presented on a mid-grey background (RGB = 128, 128, 128) (Fig. 1A). The oriented lines were phase randomised over trials, had a spatial frequency of 1.15 cycles/degree, and were shown within a circular aperture with a diameter of 5.20 degrees of visual angle. There were 4 possible line orientations for the non-target stimuli, which were used for analysis: 22.5°, 67.5° 112.5°, and 157.5°. Orange and blue lines were always shown together, rotated 45° with respect to each other, resulting in 8 unique combinations of orientations (Fig. 1B). These orientations were chosen to make sure they were orthogonal over attention conditions for the decoding analysis.

Participants were instructed to maintain fixation on a central bullseye throughout the entire experiment and to respond to a target presented at fixation using a button press response. The stimuli were presented in 64 sequences (see Fig. 1C). At the start of each sequence, participants were shown the target stimulus. The target had a specific orientation (0° or 90°) and colour (orange or blue) and was counterbalanced over sequences. Participants were instructed to attended to the lines in the target colour and ignore the lines in the other colour. The task was to press a button as soon as lines in the target orientation and colour appeared. The purpose of the task was to keep participants engaged, and to make sure participants attended to the lines of one colour, ignoring the other colour. In each sequence, the target orientation (0° or 90°) was paired with either 45° or 135° in the other colour. The target orientation could either be shown in the cued colour (target event), or in the uncued colour (foil event), and participants had to respond only to the target events. For instance, if the target was 90°-blue, the possible target stimuli for that sequence were 90°-blue/45°-orange, and 90°-blue/135°-orange, while the possible foils for that sequence were 45°-blue/90°-orange, and 135°-blue/90°-orange (see Fig. 1D).

Each sequence consisted of 104 non-target stimulus presentations. In addition, there were 1 or 2 target events and 1 or 2 foil events per sequence, counterbalanced over sequences. We added non-target padding trials at the start and end of each sequence as well as around targets and foils. The neural response of several stimulus presentations will overlap when stimulus presentations are very brief^51–55^. The stimuli at the start or end of the sequence, or those that occur after a target, might have different processing compared to typical task-related processes. The padding trials were used to capture responses related to the processing of the target, or the start or end of the sequence, and were removed from further analysis. They were randomly drawn without replacement from the 8 possible trial types (Fig. 1B). There were 4 padding trials at the start and end of each sequence, 3 padding trials before each target and foil, and 4 after. The total number of stimulus presentations in each sequence ranged between 128 and 144, depending on the number of targets and foils in each sequence. All target events, foil events, and padding were removed, and only the 104 non-target stimulus presentations in each sequence were used for further analysis.

Each stimulus in the sequence was presented for 100 ms, while the interstimulus interval (ISI) duration was either 200 ms in the constant ISI condition or varied between 100 and 300 ms in the varied ISI condition (Fig. 1C). In the varied ISI condition, there were 13 possible ISIs, equally spaced between 100 and 300 ms, reflecting the 60 Hz refresh rate of the monitor (i.e., 16.67 ms steps). We counterbalanced over sequences whether the ISI was constant or varied. Within a sequence, the 104 stimulus presentations were balanced for every combination of ISI before stimulus onset (13 for variable ISI condition) x orientation (4) x attention condition (2).

Participants completed 3 training parts during the EEG setup. Training parts 1 and 2 consisted of 4 sequences with 32 stimuli per sequence. In the first training part, participants only saw lines of the cued colour and the task was slowed down to 400 ms stimulus presentation and 400 ms ISI. In the second training part, the lines in the uncued colour were introduced, while the timing was kept the same as part 1. The third training part consisted of 8 sequences with 104 stimulus presentations per sequence. This training part was the same as the experiment: the stimulus presentation was sped up to 100 ms, and the ISI was either 200 ms (4 sequences) or varied from 100 to 300 ms (4 sequences).

### EEG acquisition and pre-processing

We recorded continuous EEG data from 64 electrodes, digitised at a sample rate of 1000-Hz, using a BrainVision ActiChamp system. The electrodes were arranged according to the international standard 10–10 system for electrode placement^56^ and were referenced online to Cz. We performed offline pre-processing using the EEGlab toolbox in Matlab^57^, following an established pre-processing pipeline^37,58^. We first filtered the data using a Hamming windowed FIR filter with 0.1 Hz high pass and 100 Hz low pass filters, re-referenced the data to an average reference, and down-sampled the data to 250 Hz. We created epochs from -100 ms to 800 ms relative to stimulus onset.

### Decoding analysis

We conducted 3 different decoding analyses, to test for (1) an effect of attention on orientation coding, (2) an effect of temporal expectation on orientation coding, and (3) an interaction between attention and temporal expectation. To investigate the time-course of the effect of attention on the coding of visual information in the brain, we collapsed the data over the different timing predictability conditions. We used MVPA to determine whether the pattern of activation across EEG channels carried information about the cued orientation and the uncued orientation for each time-point. Comparing the coding of the cued and uncued orientation allowed us to determine at which time-points an effect of attention occurred, as indicated by stronger coding of cued compared to uncued orientations. We used the CoSMoMVPA toolbox for MATLAB for all decoding analyses^59^. For decoding of the cued orientation, we trained a regularised Linear Discriminant Analysis classifier to distinguish between orientations for each time-point in the epoch. Because the cued and uncued orientations were presented simultaneously, we made sure orientations were orthogonal over attention conditions. We did this by dividing the 4 possible cued orientations into 2 pairs, 22.5° vs. 112.5° and 67.5° vs. 157.5°, and running the decoding analysis within a single pair. For instance, when the cued orientations were 22.5° and 112.5°, the uncued orientations could be either 67.5° or 157.5°, each occurring equally often for each cued orientation (Fig. 1B). We averaged decoding accuracies across pairs, resulting in a chance level of 50%. We determined decoding performance for each individual participant using a leave-one-sequence-out cross-validation approach, and then analysed the subject-averaged results at the group level. The decoding analysis of the uncued orientation was conducted in the same way. For plotting purposes, we bootstrapped 95% confidence intervals across participants using 10,000 bootstrap samples.

Note that we made sure that any difference in classification could not be driven by target-related processes in the following ways. First, we minimised effects of visual short-term memory by using a target detection task (effectively a “0-back” task), so participants did not have to retain the decoded stimulus orientation over a delay period. Participants did have to remember which was the target orientation (horizontal or vertical) for the duration of the sequence, but this could not drive the classification, as the target orientation was not informative about the decoded stimulus orientation. Critically, we only analysed the non-targets. Second, we made sure decision-making processes could not drive the classifier. Although a decision had to be made on each trial, it was a decision about whether the stimulus was a target. As we only analysed non-target trials, the decision was always the same for the analysed orientations (i.e., “not a target” decisions) and could therefore not drive the decoding. To examine the effect of temporal expectation, we split the data into the constant and variable ISI conditions, and performed the analysis described above separately for both the attention (cued or uncued) and temporal expectation (constant ISI or varied ISI) conditions, and then averaged across the cued and uncued conditions to obtain a single time-course of decoding accuracies per temporal predictability condition.

To investigate whether the effect of attention was (1) present, and (2) the same, for the two ISI conditions, we examined the orientation decoding accuracies separately for the different attention (cued or uncued) x temporal expectation (constant ISI or varied ISI) conditions.

### Statistical inference

We used Bayesian statistics to determine the evidence for above-chance decoding (alternative hypothesis) and chance decoding (null hypothesis) for each point in time^60–63^, using the Bayes Factor R package^64^. We did this for (1) the orientation coding separated for the cued and uncued conditions, (2) the orientation coding separated for the constant and varied ISI conditions, and (3) the orientation coding separated for both the attention and ISI conditions. We used a half-Cauchy prior for the alternative hypothesis to capture directional (above chance) effects. The prior was centred around chance (d = 0, i.e., 50% decoding accuracy), and had the default width of 0.707^63,65,66^. We excluded the interval ranging from d = 0 to d = 0.5 from the prior to exclude irrelevant effect sizes^67^.

We tested for an effect of attention for data combined across temporal predictability conditions, and separately for the constant and varied timing conditions, by calculating the difference between decoding accuracies for the cued and uncued orientations. Because we had the *a-priori* hypothesis that the orientation coding of the cued orientation would be higher compared to uncued orientation, we used the same half-Cauchy (directional) prior described above, centred around 0, to test whether decoding accuracies of the cued orientation were higher than for the uncued orientation. We tested for an effect of temporal expectation by comparing the orientation coding of stimuli presented in the constant and varied ISI conditions, collapsed over attention conditions. We used the half-Cauchy prior described above to test whether orientation decoding accuracies in the constant ISI condition were higher than those in the varied ISI condition. To assess whether the effect of attention was different across the two ISI conditions, we compared the difference between decoding accuracies for the cued and uncued orientations directly for the constant and varied ISI conditions. Unlike the tests described above, we did not have an *a-priori* prediction about the direction of this effect. Therefore, we used whole Cauchy (not directional) prior for the alternative and excluded an interval ranging from effect size of − 0.5 to 0.5 from the prior to make it comparable to the other analyses.

Bayes factors (BF) above 1 indicate evidence in the direction of the alternative hypothesis, and Bayes factors below 1 indicate evidence in the direction of the null hypothesis. Bayes factors below 1/3 or above 3 are usually interpreted as substantial evidence, and Bayes factors below 1/10 or above 10 are usually interpreted as strong evidence^66^. We used the conservative threshold of Bayes factors above 10 (strong evidence)^66^ to determine the onset time of above chance decoding. To avoid interpreting single time-points with evidence for above-chance decoding, when neighbouring time-points do not support this, we defined the onset time of above chance decoding as the second consecutive time-point with a Bayes factor above 10.

## Results

The purpose of the task was to make sure participants attended to the orientations in the cued colour, while ignoring the orientations in the other colour. The task was difficult due to its fast nature, ensuring participants had to pay attention. Responses within 2000 ms after the onset of the target or foil stimulus were counted as hits or false alarms respectively. Participants performed the target-detection task in the constant interval condition with a mean hit rate of 60.96% (SD = 11.60%, range = 36.73–85.42%), a mean false alarm rate on foils (target orientation in uncued colour) of 11.12% (SD = 6.46%, range = 0.00–28.26%), and a mean false alarm rate on the non-target trials of 0.34% (SD = 0.41%, range 0.00–1.68%). On the varied interval condition, participants had a mean hit rate of 58.06% (SD = 11.49%, range = 36.73–78.85%), a mean false alarm rate on foils of 9.89% (SD = 6.43, range = 0.00–25.00%), and a mean false alarm rate on non-target trials of 0.34% (SD = 0.45%, range 0.03–1.95%). This indicates that the participants were engaged in the task and managed to discriminate between the orientations of the cued and uncued colour.

**Figure 2:**
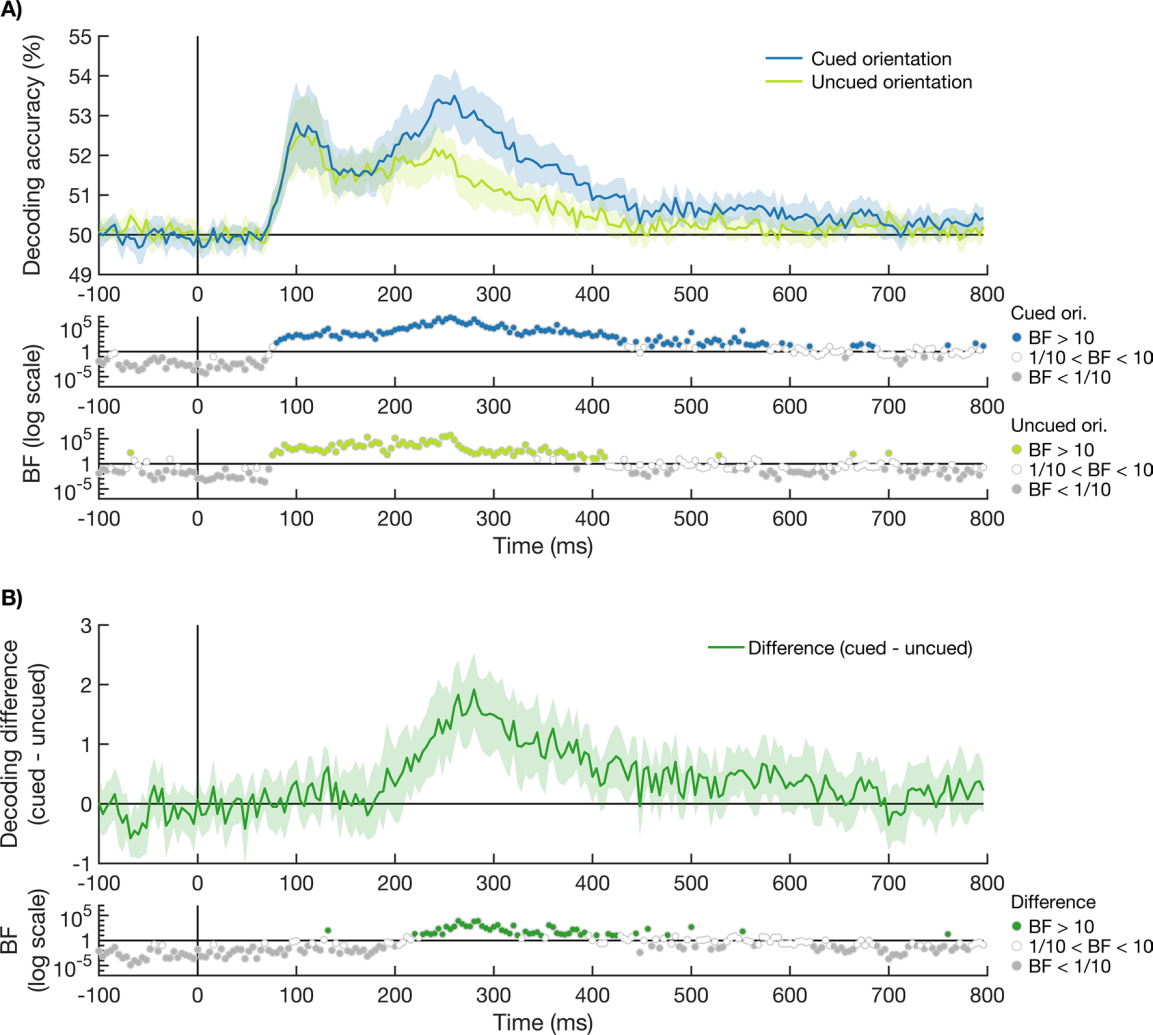
Time-course of decoding accuracies of the cued and uncued orientations. (**A**) shows the decoding accuracy of the stimulus orientation over time, when the orientation was either presented in the cued colour (blue) or uncued (light green). The stimulus onset is marked with a vertical black line at 0 ms. Theoretical chance is 50% decoding accuracy, and shaded areas around the plot lines show the bootstrapped 95% confidence interval across participants (N = 20). Bayes factors are given below the plot on a log scale. Bayes factors below 1/10 are shown in grey, indicating strong evidence for the null hypothesis. Bayes factors above 10 are shown in the plot colour (blue for cued and light green for uncued), indicating strong evidence for the alternative hypothesis. Bayes factors between 1/10 and 10 are shown in white. The cued orientation could be decoded from 84 ms after the onset of the stimulus, and the uncued orientation from 80 ms after stimulus onset. (**B**) shows the effect of attention, measured as the difference in orientation decoding between the cued and uncued orientation (cued − uncued). The Bayes factors are given below the plot on a log scale. An effect of attention was present from 232 ms after stimulus onset.

We decoded the cued and uncued orientations for each time-point to determine whether there is an effect of attention on the coding of visual information (Fig. 2A). There was strong evidence for above chance decoding of both the cued and uncued orientation from approximately 80 ms after stimulus onset (84 ms and 80 ms respectively). There was strong evidence for an effect of attention, defined as stronger coding of the cued compared to uncued orientation, from 232 ms (Fig. 2B). This suggests the brain initially represented both the cued and uncued stimuli to a similar extent, but later prioritised the processing of the attended information.

**Figure 3:**
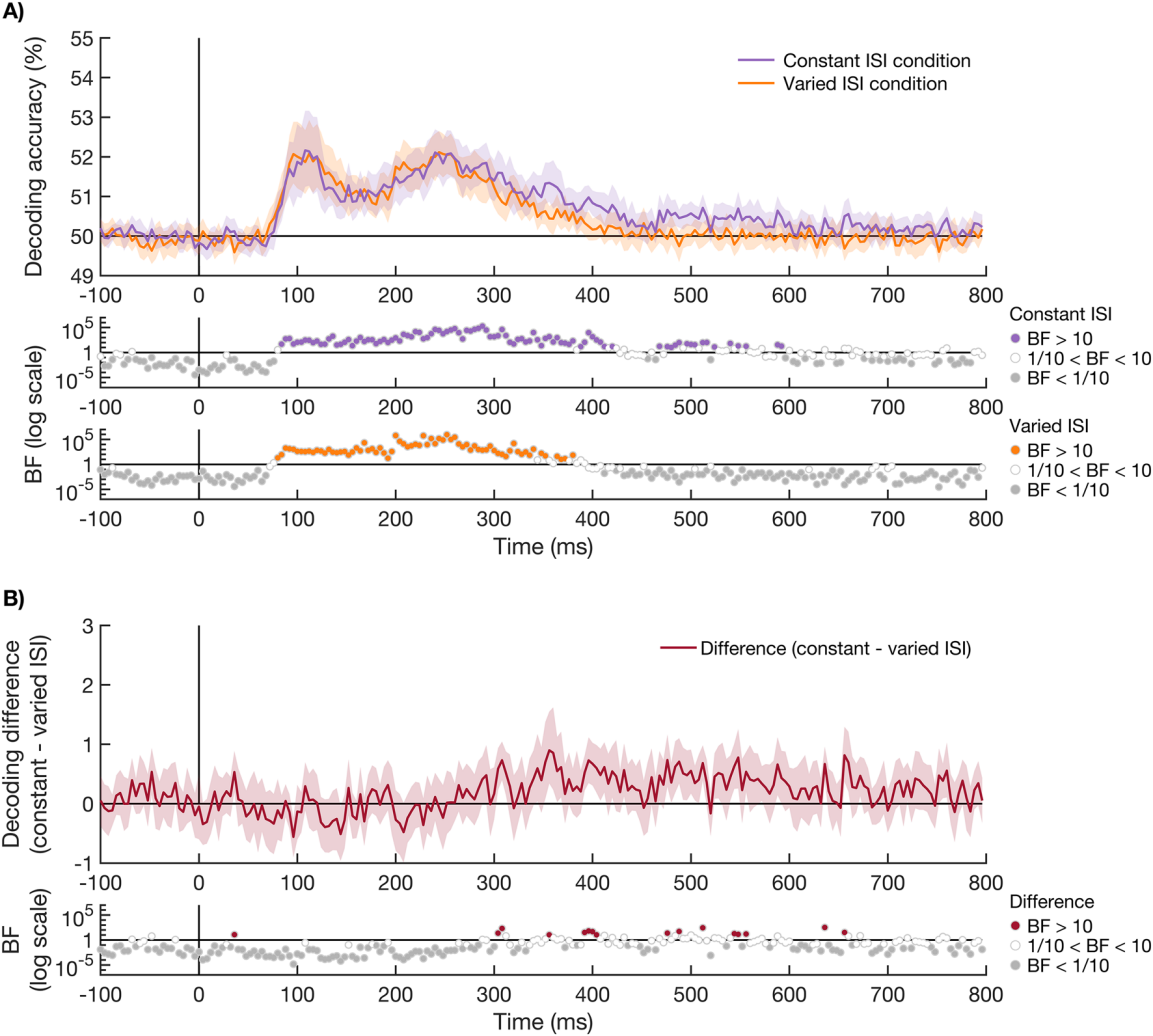
Time-course of decoding accuracies of the orientations presented at a constant and varied ISI. (**A**) shows orientation decoding accuracy of stimuli presented at a constant ISI (purple) or varied ISI (orange). Plotting conventions are the same as in Fig. 2. The orientations of stimuli presented at a constant ISI or varied ISI could be decoded from 88 and 84 ms after stimulus onset respectively. (**B**) shows the effect of temporal expectation, measured as the difference in orientation decoding between the stimuli presented at a constant or varied ISI (constant − varied ISI). The BFs are given below the plot on a log scale. There was no effect of temporal expectation on the coding of orientation information. A few time-points after 300 ms showed stronger orientation coding for the constant compared to varied ISI conditions, but the Bayes factors for most time-points in the 300 ms to 800 ms time-window either suggested inconclusive evidence or evidence for no difference.

The second aim of this study was to examine the influence of temporal expectation on the effect of attention. First, we determined whether there was a main effect of temporal expectation on the processing of visual orientation information, and then we tested whether there was an interaction between attention and temporal expectation. To determine whether there was a main effect of temporal expectation, we compared the decoding of orientation between stimuli presented in the constant and varied ISI sequences, collapsing across attention conditions. The decoding accuracies for the constant and varied ISI conditions are shown in Fig. 3A. We calculated the effect of temporal expectation as the difference between orientation coding for stimuli in the constant and varied ISI sequences (see Fig. 3B). There was no reliable effect of temporal expectation on the processing of visual information. Although a few time-points after 300 ms showed evidence for stronger orientation coding for the constant compared to varied ISI conditions (BF_10_ > 10), most time-points after 300 ms showed either inconclusive evidence or strong evidence for no effect (BF_10_ < 1/10).

**Figure 4:**
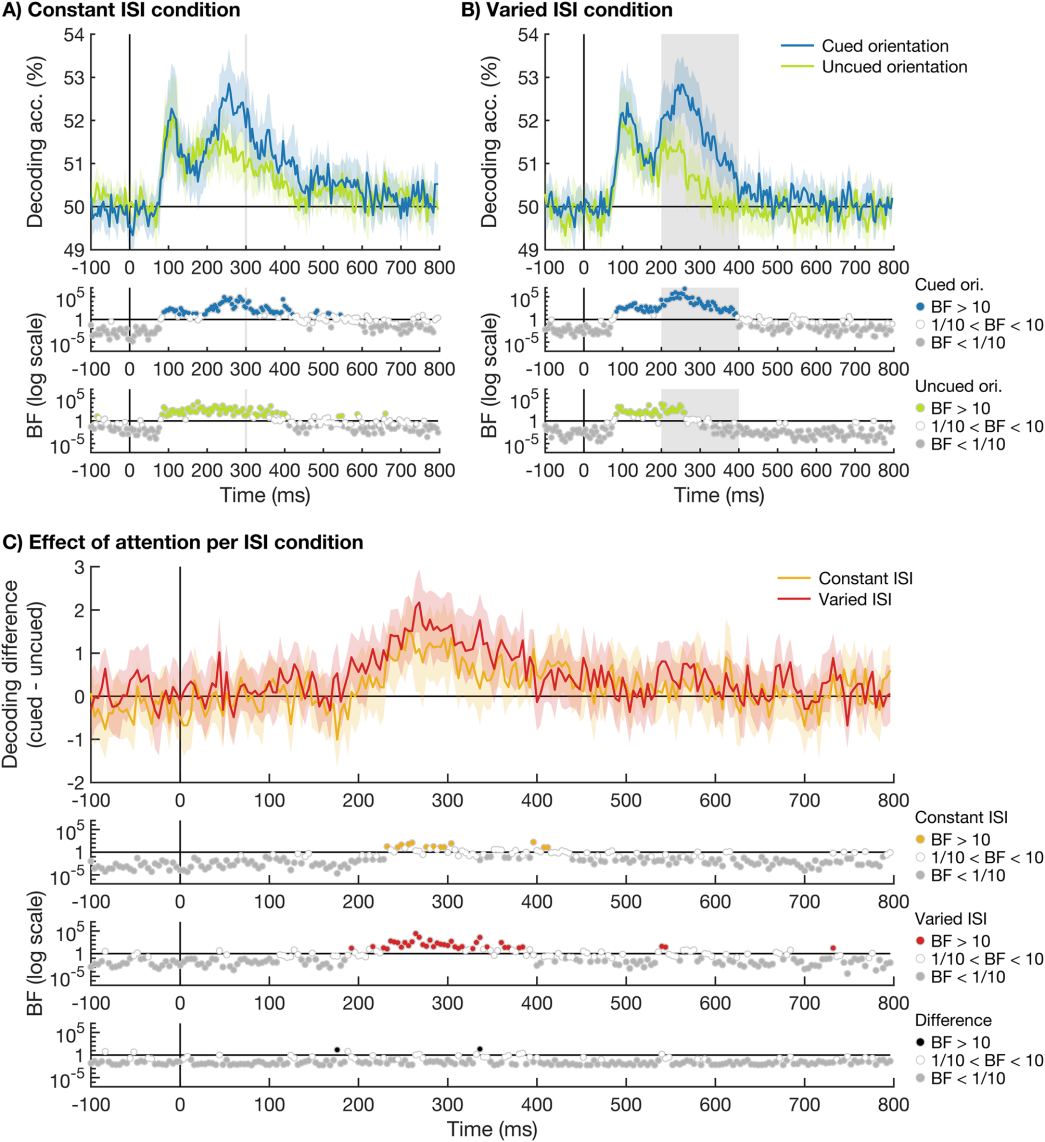
Time-course of decoding accuracies of the cued and uncued orientations for the varied and constant ISI conditions. Plots show the decoding accuracies of the cued (blue) and uncued (light green) stimulus orientation over time for the constant ISI condition in (**A**) and the varied ISI condition in (**B**). The possible onset time(s) of the next stimulus in the sequence are indicated by a grey shaded line at 300 ms in (**A**) and a grey shaded area between 200 and 400 ms in (**B**). (**C**) shows the effect of attention on stimulus coding, calculated as the difference in decoding accuracy for the cued and uncued conditions, for the constant ISI condition (yellow) and the varied ISI condition (red). We observed an effect of attention for both timing conditions, starting at 248 ms after stimulus onset for the constant ISI condition, and at 232 ms after stimulus onset for the varied ISI condition. We directly compared the effect of attention for the 2 different ISI conditions and found no 2 consecutive time-points with BF_10_ > 10. Plotting conventions are the same as in Fig. 2.

To examine whether an effect of attention was present regardless of temporal expectation, we compared the decoding of the cued and uncued orientations separately for the constant ISI sequences (Fig. 4A) and the varied ISI sequences (Fig. 4B). We observed an effect of attention for both timing conditions, starting at 248 ms after stimulus onset for the constant timing condition, and at 232 ms after stimulus onset for the varied timing condition. We directly compared the effects of attention in the two different timing conditions, to examine the interaction between attention and temporal expectation (Fig. 4C). Although a few time-points showed evidence for a larger effect of attention in the varied ISI condition, no two consecutive time-points showed strong evidence (BF_10_ > 10) for this difference, and the majority of time-points showed strong evidence for no effect (BF_10_ < 1/10). Note that although the visual input was perfectly matched between the constant and varied ISI conditions until 200 ms after stimulus onset, this is not the case after this time. While the next stimulus in the sequence was always presented 300 ms after the preceding one in the constant ISI condition, it was presented between 200 and 400 ms after stimulus onset in the varied ISI condition (see grey line in Fig. 4A and grey shaded area in Fig. 4B). This means that masking by the next stimulus in the sequence occurred earlier in some of the varied ISI trials (and later in others) compared to the constant ISI trials.

## Discussion

In this experiment we investigated the time-course of the effect of feature-based attention on visual information processing. We controlled for effects of target related processes, such as visual short-term memory and decision-making, in the following ways. We controlled for visual short-term memory effects by using a target detection task where participants did not have to retain the cued stimulus orientation over a delay period. We made sure decision-making processes could not drive the classifier by making the decision (i.e., target or not a target) orthogonal to the decoded stimulus orientations. Critically, we only analysed the non-target stimulus presentations. In addition, we investigated whether temporal expectation modulated the effects of attention on information processing by presenting the stimuli with either a constant (predictable) interval between stimuli of 200 ms, or a varied (unpredictable) interval between 100 and 300 ms. The results showed that whereas the cued and uncued visual input was initially coded equally from about 80–84 ms after stimulus onset, an effect of attention emerged from approximately 232 ms. After this time, the cued visual information was more strongly coded than uncued information. We observed this effect of attention, with a similar time-course, in both the varied and constant ISI conditions. These findings show that attention selectively enhances the relevant compared to irrelevant visual information, which is consistent with the role of attention in boosting and maintaining task-relevant information. Importantly, this effect of attention on stimulus coding occurred even when we controlled for target-related effects and occurred regardless of whether the onset timing was predictable or not.

Our findings of an effect of attention on orientation coding are consistent with previous studies that used time-resolved methods in combination with MVPA. These studies found more sustained coding for the cued compared to uncued features and/or objects^36,37,39^. In line with our previous work^39^, we show that these effects are not contingent on confounding stimulus and decision. The current study builds on previous findings by showing that feature-based attention effects occur even when we control for target-related processing and ensure that decisions do not confound the stimulus classification, and that they occur regardless of whether the stimulus onset timing is expected or not. One difference with previous research is how long the cued information was maintained in the neural signal, as this appears to be sustained for a shorter period in this study compared to previous findings^36,37,39^. This difference could be caused by a number of different processes. First, the masking of each stimulus by the subsequent stimulus occurs earlier in this paradigm compared to previous experiments^36,37,39,40^, and masking has been shown to affect the coding of visual information^51^. Secondly, this difference could be driven by the reduced requirement to maintain or manipulate the cued visual information, as this was a relatively easy target detection task. These different explanations are not mutually exclusive, and both seem likely to play a role. Although the decoding duration of cued information might be dependent on the study design, the effect of attention emerges at a similar time across studies, approximately 200–300 ms after stimulus onset. The similar timing of the emergence of preferential processing of the relevant compared to irrelevant information is in line with this effect being driven by attention. Our study adds to previous findings by unequivocally demonstrating the temporal dynamics of attention on the processing of visual information, without target-related processing confounds. In addition, our design allows us to study attended and unattended stimuli presented simultaneously in the same spatial location, in a fast and efficient paradigm.

In this study, we observed preferential coding of the attended information over the distractor information after 200 ms, suggesting that the attentional selection of the relevant colour feature biased visual processing at this time. A change in decoding could be driven by different factors. For instance, it could be driven by a reduction in trial-to-trial variability in the neural response, or by a gain in the neural coding of the stimulus features in the attended condition. The time-course found in this study suggests a top-down bias of attention on visual processing, in line with a large body of literature on non-human primates^1,68–71^ as well as humans^72–76^. The onset time of our attention effect is consistent with previous univariate work, which found effects of feature-based attention after 200 ms at anterior sites and 225 ms at posterior sites^77–79^. In addition, the timing is similar to the N2pc component, associated with the selection of relevant information, and the distractor positivity (P_D_) component, associated with the suppression of irrelevant information, both of which are observed around 200 ms after stimulus onset in visual search studies^80–85^. Our results show that the coding of the attended information is maintained after 200 ms, whereas the stimulus coding of the unattended information decreases after 200 ms. This could reflect the selection of the relevant information for further processing and/or the suppression of the distracting information and could therefore be associated with the N2PC and P_D_ components. The time-course of feature-based attention we have identified differs from univariate spatial attention studies, which found that spatial attention modulations can occur as early as 70-100 ms after stimulus onset^25–27,86–90^. This difference is likely due to the necessity of some initial stimulus processing to identify the relevant feature before feature-based attention can be applied. Spatial attention, on the other hand, can be allocated to the location where the stimulus is likely to appear, even before the stimulus is presented^16,91,92^. Here we looked at only attention to colour as a representation of feature-based attention, it is possible that processing of other features might differ slightly in time-course. The current work adds to the univariate findings by revealing what happens to both the attended and distractor information over time, even when both stimuli are presented simultaneously at the same location.

We found evidence that temporal expectation did not interact with the effect of attention on visual processing for the timescale used in this paradigm: a constant ISI of 200 ms and a varied ISI of 100–300 ms. The stimulus presentation duration was kept constant at 100 ms, resulting in a stimulus onset asynchrony of 300 ms, and 200–400 ms respectively. This finding fits with findings from Smout and colleagues, who showed that expectation about stimulus identity did not interact with the effect of attention on stimulus coding. Our study adds to these findings by investigating the contribution of temporal expectation. It is possible that an interaction would occur when temporal expectations are violated, in line with the findings of Smout and colleagues^40^, who found an interaction between attention and expectation about stimulus features on the processing of mismatch information only. Future work could therefore compare a predictable timing condition to a condition where temporal expectation is violated, rather than a condition where there is no temporal expectation as in our varied condition.

We did not find a main effect of temporal expectation on orientation processing. Although a few time-points after 300 ms showed evidence for stronger orientation coding in the constant compared to varied ISI condition, likely driven by more sustained decoding of the uncued orientation in the constant condition, most time-points showed either inconclusive evidence or evidence for no difference. The difference in those few time-points is likely attributable to the difference in visual stimulation starting after 200 ms. The next stimulus, which is likely to cause masking, occurred earlier in some of the varied ISI trials compared to the constant ISI trials. One possible interpretation of the lack of an effect is that there must be certainty about the stimulus feature in order to prioritise processing a certain window in time. While the general shape and colours of the stimuli in our paradigm were expected, the task-relevant feature (orientation) was not. Another possible interpretation is that temporal expectation might not affect the early sensory response, but rather influences the later ‘decision’ stages of information processing^93^. Several studies support this possibility, although these studies investigated expectations about spatial location^42^, stimulus features^40,48,94^ and motor responses^48^ rather than temporal expectations. However, the lack of a main effect of temporal expectation does not seem in agreement with other studies that found evidence for enhanced visual processing for stimuli presented at a constant compared to varied ISI^49,50^. One possibility for this difference is the difference in analysis methods. Whereas we used MVPA of EEG data to track the coding of visual information, previous studies have investigated the effect of implicit temporal expectations on phase-locking of oscillations^49^ and behaviour^49,50^. This means that our results are not directly comparable. Another possible explanation for the apparent difference between our results and other studies of the effects of temporal expectation^49,50^ could be the timescale, as other studies have used a slower ISI in the predictable timing condition (400 ms compared to 200 ms), as well as a wider range of range of possible ISIs for the unpredictable timing condition (200–600 ms instead of 100–300 ms)^49,50^. It is possible that this, perhaps stronger, manipulation of temporal expectation would lead to different results in our paradigm, as this would give participants more time to process the current stimulus and prepare for the next stimulus. Another, not mutually exclusive, explanation is that the perceptual difficulty of the stimuli plays a role in whether temporal attention can affect the processing of visual information. Although our task was difficult due to its fast nature, the *perceptual* difficulty was low, as the non-targets were always rotated at least 22.5° from the target orientation and there was no added visual noise. This was not the case for some of the previous studies, where target orientations were embedded in visual noise^49,50^. It is possible that the effect of temporal expectation may have a larger effect under conditions of high perceptual difficulty, when there is more to gain from attending precisely in time. Future work could disentangle whether temporal expectation effects are dependent on perceptual difficulty and longer time scales.

It is also important to consider different types of expectations. This study focused on expectations of *when* something will happen, which we term temporal expectation, but expectations about upcoming events often combine ‘when’ with ‘what’ and ‘where’. Some studies have found that temporal expectation can interact with spatial expectation^95^, as well as expectation about object features^96^. These results show that different types of expectation can work together, with the largest effects when participants have an expectation about both *where* or *what* will appear and *when*. It is therefore important to note that in this study, participants had a spatial expectation about where the stimulus would appear, as this was kept constant. In addition, participants had an idea about the general shape and colours of the stimulus, but no expectation about what the orientation of the upcoming stimulus was. We therefore need to interpret the results of the temporal expectation manipulation keeping in mind there was also a spatial expectation and an object feature expectation about colour, but no object feature expectation about orientation. This could mean that an expectation about the relevant stimulus feature might be needed to observe an effect of temporal expectation on stimulus coding.

Taken together, our results showed that attention selectively enhanced the high-level processing of stimulus relevant features from about 232 ms after stimulus onset, without target-related processing confounds. The selection of task-relevant information occurred even when the stimulus presentation was fast and occurred regardless of whether the onset was temporally predictable or not. These findings reveal a detailed picture for the time-course of the prioritisation of task-relevant information, in the presence of competing information at the same time and location.

## Data Availability

The raw and pre-processed data are available via OpenNeuro (https://openneuro.org/datasets/ds004043), and the analysis code is available via the Open Science Framework (https://doi.org/10.17605/OSF.IO/5B8K6).
